# Diagnosis of enteropathy‐associated T‐cell lymphoma (EATL) on peripheral blood: A pleiomorphic morphology and an unusual immunophenotype

**DOI:** 10.1002/jha2.733

**Published:** 2023-06-17

**Authors:** Léa Ousset, François Vergez, Camille Laurent, Lucie Oberic, Alban Canali

**Affiliations:** ^1^ Haematology Laboratory Cancer University Institute of Toulouse – Oncopole Toulouse France; ^2^ Department of Pathology Cancer University Institute of Toulouse – Oncopole Toulouse France; ^3^ Department of Haematology Cancer University Institute of Toulouse – Oncopole Toulouse France

**Abbreviation**
EATL: enteropathy‐associated T‐cell lymphoma

1


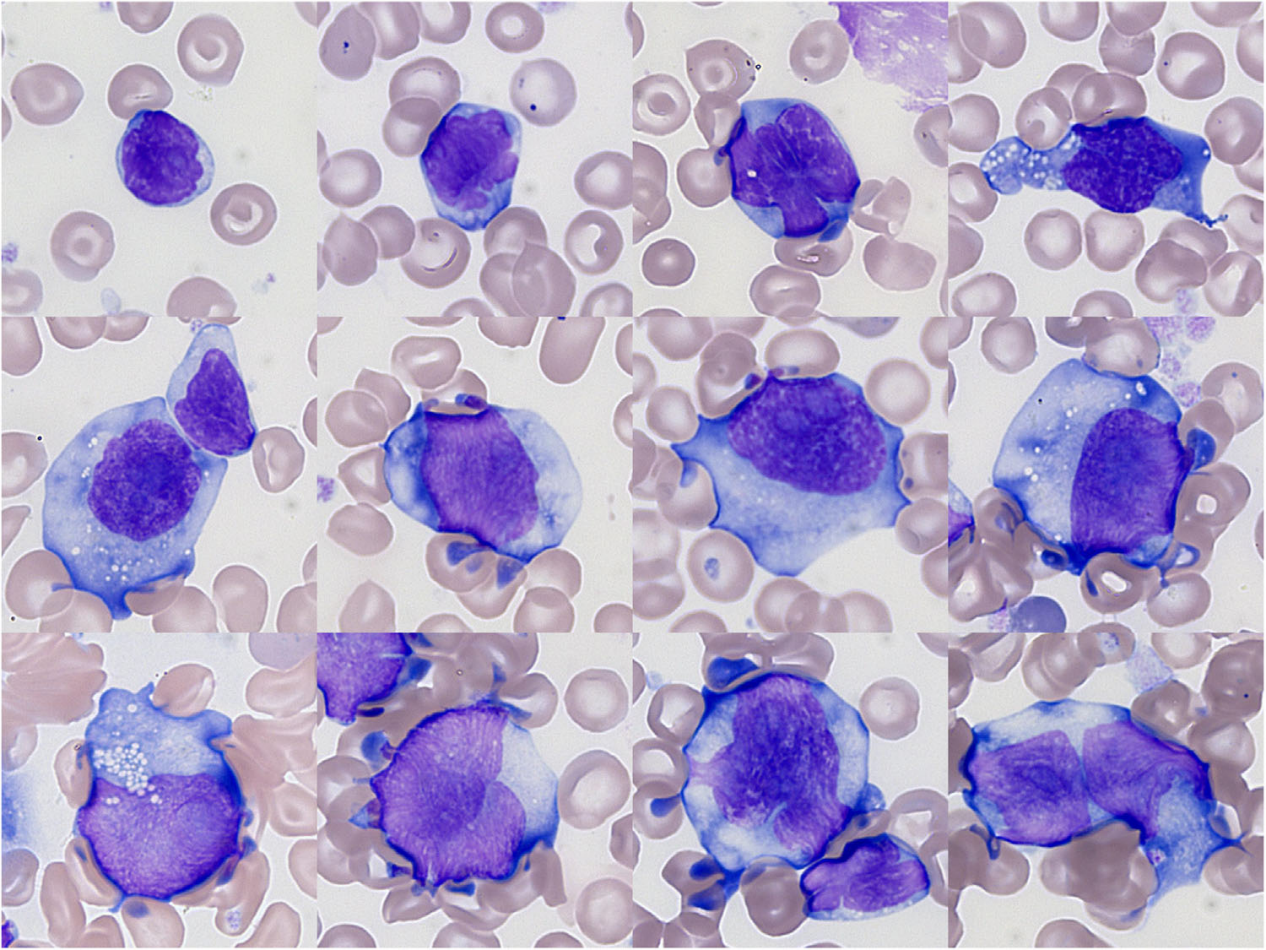


A 72‐year‐old woman known for coeliac disease for several years presented with abdominal pain and deterioration of the general status. Blood biochemistry revealed a significant tumor lysis syndrome which led to her transfer to the intensive care unit. Her full blood count showed anemia (haemoglobin concentration 107 g/L), and leucocytosis (WBC 156 × 10^9^/L). Microscopic examination of the blood film showed 86% pleiomorphic lymphomatous cells (all images May–Grünwald–Giemsa, ×100 objective) which may have been underestimated because of many apoptotic or smudge cells. These cells formed a continuum from small elements with high nucleocytoplasmic ratio, irregular nucleus, decondensed chromatin, and hyperbasophilic cytoplasm to very large elements with low nucleocytoplasmic ratio, irregular nucleus, more immature chromatin with indistinct nucleoli, and abundant cytoplasm with similar hyperbasophilic appearance and sometimes small vacuoles.

Flow cytometry immunophenotyping showed an atypical profile with a very strong CD45, membrane CD3m− but cytoplasmic CD3c+, CD2−, CD5−, CD7+/−, CD4−, CD8−, TCRαβ−, TCRγδ−, CD56−, CD103−, and CD30+. The complex and monosomal karyotype showed, among many abnormalities, deletion of the long arm of chromosome 16 between bands 16q11 and 16q13 as described in the literature. Next‐generation sequencing showed *TET2* and *EP300* mutations of undetermined significance (VAF = 13.72% and 7.72%, respectively). Duodenal and stomach biopsy revealed slightly dystrophic mucosa with mild atrophy and a site of high‐grade intestinal T‐cell lymphoma infiltration. Tumor cells consisted of medium to large pleomorphic cells with prominent nucleoli. They were strongly CD3+ and negative for other T cell markers including TCR expression. They expressed cytotoxic markers (TIA1 and perforin), and some large cells were positive for CD30. The Ki67 index was around 95%. Diagnosis of enteropathy‐associated T‐cell lymphoma (EATL) was therefore confirmed. Despite all the care provided by the medical unit, the patient passed away 4 days later.

The diagnosis of EATL is a challenging situation due to its rarity. Although the circulating form is even rarer, knowledge of these unusual morphological and immunophenotypic features can speed up diagnosis by directing the clinician to a histological examination of the digestive tract, and enable treatment to be initiated as early as possible.

It is therefore important for biologists to know the association of adult‐onset coeliac disease with these atypical circulating cells, both morphologically and immunophenotypically. The absence of CD3m and most T‐lineage markers on lymphoma cells should lead to a search for CD3c and additional markers, such as CD30 in this case.

## AUTHOR CONTRIBUTIONS

Ousset, Vergez, and Canali wrote the paper. Ousset and Vergez performed the blood smear examination. Vergez and Canali performed flow cytometric studies. Canali took the pictures. Laurent performed histological and molecular studies. Oberic followed the patient. All authors contributed to the final manuscript.

## FUNDING STATEMENT

This study was funded by Centre Hospitalier Universitaire de Toulouse.

## CONFLICT OF INTEREST STATEMENT

The authors have no conflict of interest.

## ETHICS APPROVAL STATEMENT

This manuscript respects the ethic policy of CHU Toulouse for the treatment of human research participants.

## PATIENT CONSENT STATEMENT

This manuscript respects the ethic policy of CHU Toulouse for the treatment of human research participants. The authors did not obtain written informed consent from the patient but the patient did not object to his data being used for research purposes (as required by ethic policy of CHU Toulouse). Written permission for reproduction from the copyright owners will be provided if the submission is accepted.

## Data Availability

Data sharing does not apply to this article as no new data were created or analyzed in this study.

